# Retraction: Relay cropping of cotton in wheat improves productivity of cotton-wheat cropping system

**DOI:** 10.1371/journal.pone.0272188

**Published:** 2022-08-03

**Authors:** The PLOS ONE Editors

The *PLOS ONE* Editors retract this article [1] because it was identified as one of a series of submissions for which we have concerns about authorship, competing interests, and peer review. We regret that the issues were not addressed prior to the article’s publication.

AAH, MA, and MSE either did not respond directly or could not be reached. MUA responded but expressed neither agreement nor disagreement with the editorial decision. AM, MNA, MT, and ZA did not agree with the retraction.
